# Efficacy of nasal endoscopic dacryocystorhinostomy for chronic dacryocystitis: A systematic review protocol of randomized controlled trial

**DOI:** 10.1097/MD.0000000000014889

**Published:** 2019-03-22

**Authors:** Xiao Chen, Yang Liu

**Affiliations:** a Department of Ophthalmology, Yangling Demonstration District Hospital, Yangling; b Department of Ophthalmology, Yan’an People's Hospital, Yan’an, China.

**Keywords:** chronic dacryocystitis, efficacy, nasal endoscopic dacryocystorhinostomy, randomized controlled trial, safety, systematic review

## Abstract

**Background:**

This study aims to assess the efficacy and safety of nasal endoscopic dacryocystorhinostomy (NED) for patients with chronic dacryocystitis (CD).

**Methods:**

The following 7 electronic databases will be searched from inception to the present: Cochrane Library, EMBASE, MEDLINE, Cumulative Index to Nursing and Allied Health Literature, Web of Science, Allied and Complementary Medicine Database, and Chinese Biomedical Literature Database. In addition, the clinical trials registry and reference lists of included studies will also be searched. We will only include randomized controlled trials of NED for CD in this systematic review. Two review authors independently carry out the study selection, data extraction, and methodological quality assessment. Whenever it is possible, we will pool the data and conduct meta-analysis by using RevMan 5.3 software.

**Results:**

This study will evaluate the efficacy and safety of NED for patients with CD. The primary outcome includes success rate of ostial patency. The secondary outcomes consist of duration of surgery, quality of life, postoperative complications, and surgeon's comfort.

**Conclusion:**

The findings of this study may summarize the latest evidence of NED for patients with CD.

**PROSPERO registration number:**

PROSPERO CRD42019123664.

## 1 Introduction

Chronic dacryocystitis (CD) is a common infection disorder of the lacrimal sac typically associated with nasolacrimal duct obstruction (NLDO).^[1–3]^ The cause of NLDO consists of idiopathic or secondary to various infections, inflammations, traumatic injuries, as well as the neoplasms.^[4,5]^ This condition ultimately results in stagnation of tears and accumulation of mucoid secretions, which contributes to the bacterial infections and genesis of dacryocystitis.^[6,7]^ Previous study has reported that this condition often affects adults above 30 years old, especially among female population.^[8]^

Nasal endoscopic dacryocystorhinostomy (NED) are reported to treat CD by numerous clinical trials.^[9–28]^ However, up to date, the efficacy and safety of NED for the treatment of CD is still inconclusive. Additionally, no systematic review has addressed this issue. Therefore, the protocol of this systematic review will evaluate the efficacy and safety of NED for patients with CD.

## 2 Methods

### 2.1 Study registration

This protocol study has registered with PROSPERO (CRD42019123664), and it has designed and reported according to the Preferred Reporting Items for Systematic Reviews and Meta-Analysis Protocol statement guidelines.^[29]^

### 2.2 Eligibility criteria for study selection

#### 2.2.1 Types of studies

All relevant randomized controlled trials (RCTs) regarding the NED for patients with CD will be fully considered for inclusion without any language and publication status restrictions. However, any other studies, including non-clinical trials, non-controlled studies, non-RCTs, and quasi-RCTs will be excluded.

#### 2.2.2 Types of interventions

The experimental group includes NED monotherapy. However, the studies will not be considered if the combination of NED with other treatments was utilized in the experimental group. The control group can receive any therapies, except the NED.

#### 2.2.3 Types of patients

Patients clinically diagnosed with CD will be included in this study, regardless the race, sex, and age.

#### 2.2.4 Types of outcome measurements

The primary outcome includes success rate of ostial patency. The secondary outcomes consist of duration of surgery (as recorded by minutes), quality of life (as assessed by any scales, such as Short Form Health Survey is a 36-item), postoperative complications, and surgeon's comfort (as measured by any related instruments).

### 2.3 Search strategy for identification studies

We will search the following 7 electronic databases from their inceptions to the present: Cochrane Library, EMBASE, MEDLINE, Cumulative Index to Nursing and Allied Health Literature, Web of Science, Allied and Complementary Medicine Database, and Chinese Biomedical Literature Database. Moreover, we will also search clinical trials registry and reference lists of included studies. Any relevant RCTs on assessing the efficacy and safety of NED for CD will be fully considered in this systematic review. The search strategy sample of electronic database Cochrane Library is shown in Table 1. Similar search strategy for other electronic databases will also be built and applied.

**Table 1:**
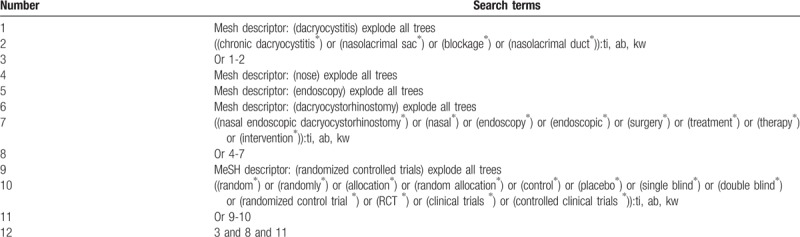
Search strategy for Cochrane Library.

### 2.4 Data collection and analysis

#### 2.4.1 Study selection

**Figure 1:**
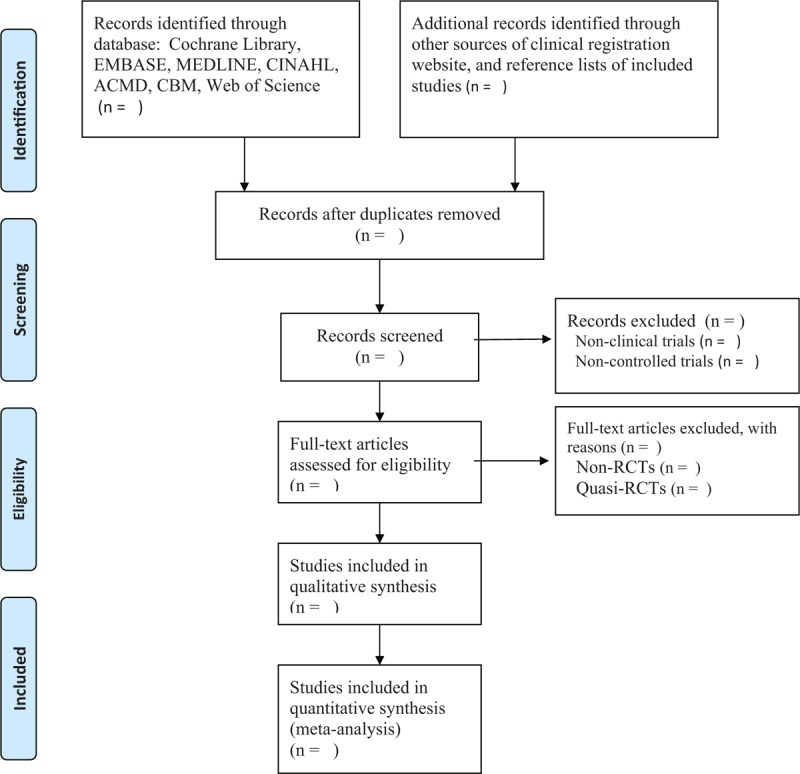
Process of study selection.

Two review authors will independently scan the title, and abstract for each study according to the predefined eligibility criteria. The full texts will be read if there is insufficient information to judge a study from the title and abstract. Any disagreements regarding the study selection between 2 review authors will be resolved by consulting a third review author. The whole process of study selection is shown in Figure 1.

#### 2.4.2 Data collection and management

Two review authors will independently extract the data according to the predefined data extraction sheet. It will consist of study characteristics, patient characteristics, and study methods, details of treatments in both experimental and control groups, and all outcome measurements. Any divisions regarding the data extraction will be solved by a third review author through discussion.

#### 2.4.3 Risk of bias assessment for included studies

Two review authors will independently assess the methodological quality for each included study by using the Cochrane risk of bias tool. It comprises of 7 domains. Each domain will be judged as low risk of bias, unclear risk of bias, or high risk of bias. Any divergences regarding the methodological assessment will be resolved by a third review author through discussion.

#### 2.4.4 Treatment effect measurement

In this study, the continuous data will be expressed as mean difference and 95% confidence intervals (CIs), while the dichotomous data will be expressed as risk ratio and 95% CIs.

#### 2.4.5 Missing data dealing with

If there is missing data or insufficient information, we will contact the primary corresponding authors to require them. If those data is unachievable, we will only analyze the available data, and will discuss its impacts.

#### 2.4.6 Heterogeneity assessment

We will use *I*^*2*^ test to identify the heterogeneity. Reasonable heterogeneity will be considered if *I*^*2*^ ≤50%. Otherwise, significant heterogeneity will be regarded if *I*^*2*^ >50% is identified.

#### 2.4.7 Data synthesis

If *I*^*2*^ ≤50%, a fixed-effect model will be used to pool the data and meta-analysis will be conducted. If *I*^2^ >50%, a random-effect model will be used, and data pooled and meta-analysis will be carried out according to the results of the subgroup analysis. Under such situation, subgroup analysis will be conducted. If there is still substantial heterogeneity after the subgroup analysis, then data will not be pooled and meta-analysis will not be performed. Instead, a narrative summary will be described.

#### 2.4.8 Subgroup analysis

Subgroup analysis will be carried out to detect any possible reasons that may cause significant heterogeneity. It will be conducted in accordance with the different characteristics, types of experimental and control interventions, or the outcome measurement instruments.

#### 2.4.9 Sensitivity analysis

Sensitivity analysis will be conducted to check the robustness and stability of pooled outcome results, methodological quality, and the potential impacts of missing data.

#### 2.4.10 Reporting bias

If more than 10 qualified RCTs are included, we will conduct the funnel plot^[30]^ and Egg regression test to check the possible reporting bias.^[31]^

## 3 Discussion

Researchers hypothesize that NED plays an important role in the treatment of patients with CD. However, until currently, only literature on the efficacy and safety of NED for CD has been conceptual. Given emerging clinical trials on NED for CD have reported, we aim to carry out a systematic research synthesis to inform the efficacy and safety of NED for CD. We are expected to establish the current knowledge base regarding the efficacy and safety of NED for the treatment of CD. The findings of this study will be disseminated to a variety of stakeholders interested in NED treatment to inform both the researchers for further studies and clinicians focusing on the public health approach to education.

## Author contributions

**Conceptualization:** Xiao Chen, Yang Liu.

**Data curation:** Xiao Chen, Yang Liu.

**Formal analysis:** Xiao Chen.

**Funding acquisition:** Yang Liu.

**Investigation:** Yang Liu.

**Methodology:** Xiao Chen.

**Project administration:** Yang Liu.

**Resources:** Xiao Chen, Yang Liu.

**Software:** Xiao Chen.

**Supervision:** Yang Liu.

**Validation:** Xiao Chen, Yang Liu.

**Visualization:** Xiao Chen, Yang Liu.

**Writing – original draft:** Xiao Chen, Yang Liu.

**Writing – review and editing:** Xiao Chen, Yang Liu.

## References

[1] Islam MR Wadud SA Akhanda AH Outcome of transcanalicular endolaser and external dacryocystorhinostomy in chronic dacryocystitis. Mymensingh Med J 2018;27:673–8. 30487479

[2] Meireles MN Viveiros MM Meneghin RL Dacryocystectomy as a treatment of chronic dacryocystitis in the elderly. Orbit 2017;36:419–21. 28816565 10.1080/01676830.2017.1353111

[3] Tang LF Qin G Yang YC Therapeutic effect of two kinds of surgical treatment for chronic dacryocystitis: external dacryocystorhinostomy and endoscopic dacryocystorhinostomy. Lin Chung Er Bi Yan Hou Tou Jing Wai Ke Za Zhi 2017;31:1029–31. 29798171 10.13201/j.issn.1001-1781.2017.13.016

[4] Mills DM Bodman MG Meyer DR The microbiologic spectrum of dacryocystitis: a national study of acute versus chronic infection. Ophthal Plast Reconstr Surg 2007;23:302–6. 10.1097/IOP.0b013e318070d237 17667103

[5] Sanmartin ZJ Dacriocistografia com subtração digital (DGGSD). Arq Bras Oftalmol 1998;61:224–8.

[6] Amin RM Hussein FA Idriss HF Pathological, immunohistochemical and microbiologicalal analysis of lacrimal sac biopsies in patients with chronic dacrocystitis. Int J Ophthalmol 2013;6:817–26. 24392331 10.3980/j.issn.2222-3959.2013.06.14 PMC3874522

[7] Anderson NG Wojno TH Grossniklaus HE Clinicopathologic findings from lacrimal sac biopsy specimens obtained during dacryocystorhinostomy. Ophthalmic Plast Reconstr Surg 2003;19:173–6. 12918549 10.1097/01.iop.0000066646.59045.5a

[8] Bharathi MJ Ramakrishnan R Maneksha V Comparative bacteriology of acute and chronic dacryocystitis. Eye (Lond) 2008;22:953–60. 17603466 10.1038/sj.eye.6702918

[9] Wang G Wei W Song Y Effect of nasal endoscopic dacryocystorhinostomy on chronic dacryocystitis. J Hubei Univ Sci Technol 2018;6:505–6.

[10] Wang G Wei W Song Y Observation on the effect of nasal endoscopic dacryocystorhinostomy in the treatment of chronic dacryocystitis. J Hubei Univ Sci Technol 2018;32:505–6.

[11] Qin ZY Liang ZJ Luo YJ Clinical observation of sinus endoscopic dacryocystorhinostomy for chronic dacryocystitis. Chin J Ophthalmol 2018;8:170–4.

[12] Li JJ Nasal endoscopic dacryocystorhinostomy for the treatment of chronic dacryocystitis. World's Latest Med Inf Digest 2017;17:223.

[13] Liu Hx Gao P Jin W Clinical observation of nasal endoscopic dacryocystorhinostomy for chronic dacryocystitis. J Clin Ophthalmol 2016;24:367–8.

[14] He LY Clinical analysis of 45 cases of chronic dacryocystitis treated by nasal endoscopic dacryocystorhinostomy. Yunnan Med J 2015;36:525–7.

[15] Luo ZY Yang XQ Li L Clinical observation of nasal endoscopic dacryocystorhinostomy for chronic dacryocystitis. Sichuan Med J 2014;35:833–4.

[16] Guo AH Li L Observation of the effect of nasal cavity endoscopic dacryocystorhina in the treatment of chronic dacryocystitis with nasolacrimal duct obstruction. J Ningxia Med Univ 2014;36:704–6.

[17] Huang Z Therapeutic effect of nasal endoscopic lacrimal sac and combined drainage stent implantation in the treatment of chronic dacryocystitis. Modern Diagn Treat 2014;25:1705–7.

[18] Zhou Q Therapeutic effect of nasal endoscopic dacryocystorhinostomy for chronic dacryocystitis. J Med Forum 2014;35:138–9.

[19] Zhang WD Therapeutic effect of nasal endoscopic dacryocystorhinostomy for chronic dacryocystitis. Chin Foreign Med Care 2014;33:42–3.

[20] Xu WJ Yang J Clinical observation of nasal dacryocystorhinostomy with dacryocystorhinostomy. Chin Med Sci 2012;2:195–7.

[21] Wei YJ Liu XH Shi YM Nasal endoscopic dacryocystorhinostomy for chronic dacryocystitis. Chin J Sch Doctor 2012;26:556.

[22] Sun DY Clinical observation of nasal cavity endoscopic dacryocystorhinostomy for chronic dacryocystitis. Jilin Med J 2012;33:83–4.

[23] Zhao SF Zhu YZ Zhang JP Clinical experience of nasal endoscopic dacryocystorhinostomy in the treatment of chronic dacryocystitis. Ningxia Med J 2011;33:1233–4.

[24] Zeng YF Liang LL Chen LM Therapeutic effect of nasal endoscopic dacryocystorhinostomy for chronic dacryocystitis. Pract Clin Med 2011;12:85.

[25] Cai T Clinical observation of treatment of chronic dacryocystitis with sinus endoscopic dacryocystorhinostomy. China Pract Med 2010;5:101–2.

[26] Li Tao Endoscopic nasal lacrimal sac sac surgery for the treatment of chronic dacryocystitis in 123 cases. Med Inf 2009;22:1159–60.

[27] Xi W Cui W Comparison of nasal endoscopic dacryocystorhinostomy combined with nasal lacrimal sac anastomosis in the treatment of chronic dacryocystitis. Shaanxi Med J 2009;38:499.

[28] Chen XQ Chen FX Zhao Y Therapeutic effect of nasal endoscopic dacryocystorhinostomy for chronic dacryocystitis. Int J Ophthalmol 2007;3:848–9.

[29] Moher D Shamseer L Clarke M Preferred reporting items for systematic review and meta-analysis protocols (PRISMA-P) 2015 statement. Syst Rev 2015;4:1. 25554246 10.1186/2046-4053-4-1 PMC4320440

[30] Sutton AJ Duval SJ Tweedie RL Empirical assessment of effect of publication bias on meta-analyses. BMJ 2000;320:1574–7. 10845965 10.1136/bmj.320.7249.1574 PMC27401

[31] Egger M Davey Smith G Schneider M Bias in meta-analysis detected by a simple, graphical test. BMJ 1997;315:629–34. 9310563 10.1136/bmj.315.7109.629 PMC2127453

